# Protocol for the Digital, Individualized, and Collaborative Treatment of Type 2 Diabetes in General Practice Based on Decision Aid (DICTA)—A Randomized Controlled Trial

**DOI:** 10.3390/nu17152494

**Published:** 2025-07-30

**Authors:** Sofie Frigaard Kristoffersen, Jeanette Reffstrup Christensen, Louise Munk Ramo Jeremiassen, Lea Bolette Kylkjær, Nanna Reffstrup Christensen, Sally Wullf Jørgensen, Jette Kolding Kristensen, Sonja Wehberg, Ilan Esra Raymond, Dorte E. Jarbøl, Jesper Bo Nielsen, Jens Søndergaard, Michael Hecht Olsen, Jens Steen Nielsen, Carl J. Brandt

**Affiliations:** 1 Steno Diabetes Center Odense, Odense University Hospital, 5000 Odense, Denmark; jsoendergaard@health.sdu.dk (J.S.); jsn@rsyd.dk (J.S.N.); cbrandt@health.sdu.dk (C.J.B.); 2 Department of Clinical Research, University of Southern Denmark, 5230 Odense, Denmark; 3 Research Unit of General Practice, Department of Public Health, University of Southern Denmark, 5230 Odense, Denmark; jrchristensen@health.sdu.dk (J.R.C.); lramo@health.sdu.dk (L.M.R.J.); lkylkjaer@health.sdu.dk (L.B.K.); nfroelich@health.sdu.dk (N.R.C.); swehberg@health.sdu.dk (S.W.); djarbol@health.sdu.dk (D.E.J.); jbnielsen@health.sdu.dk (J.B.N.); 4 Research Unit of General Practice, 8000 Aarhus, Denmark; 5 DRIVEN, Institute of Sports Science and Clinical Biomechanics, University of Southern Denmark, 5230 Odense, Denmark; 6 Research Unit of User Perspectives and Community-Based Interventions, Department of Public Health, University of Southern Denmark, 5230 Odense, Denmark; swj@ph.au.dk; 7 Department of Public Health, Faculty of Health, 8000 Aarhus, Denmark; 8 Center of General Practice, University of Aalborg, 9220 Aalborg, Denmark; jkk@dcm.aau.dk; 9 Department of Cardiology, Amager and Hvidovre Hospital, 2650 Copenhagen, Denmark; ilan.esra.raymond@regionh.dk; 10 Department of Internal Medicine 1 and Steno Diabetes Center Zealand, Holbæk Hospital, 4300 Holbæk, Denmark; michael.olsen@dadlnet.dk; 11 Department of Clinical Medicine, University of Copenhagen, 2200 Copenhagen, Denmark; 12 Liva Healthcare, 1434 Copenhagen, Denmark

**Keywords:** type 2 diabetes, digital health, lifestyle coaching, patient-reported outcomes, clinical decision support, health behavior change, interactive advice, participant engagement, quality of life, weight loss

## Abstract

**Background**: Despite significant advancements in diabetes care, many individuals with type 2 diabetes (T2D) do not receive optimal care and treatment. Digital interventions promoting behavioral changes have shown promising long-term results in supporting healthier lifestyles but are not implemented in most healthcare offerings, maybe due to lack of general practice support and collaboration. This study evaluates the efficacy of the Digital, Individualized, and Collaborative Treatment of T2D in General Practice Based on Decision Aid (DICTA), a randomized controlled trial integrating a patient-centered smartphone application for lifestyle support in conjunction with a clinical decision support (CDS) tool to assist general practitioners (GPs) in optimizing antidiabetic treatment. **Methods**: The present randomized controlled trial aims to recruit 400 individuals with T2D from approximately 70 GP clinics (GPCs) in Denmark. The GPCs will be cluster-randomized in a 2:3 ratio to intervention or control groups. The intervention group will receive one year of individualized eHealth lifestyle coaching via a smartphone application, guided by patient-reported outcomes (PROs). Alongside this, the GPCs will have access to the CDS tool to optimize pharmacological decision-making through electronic health records. The control group will receive usual care for one year, followed by the same intervention in the second year. **Results**: The primary outcome is the one-year change in estimated ten-year cardiovascular risk, assessed by SCORE2-Diabetes calculated from age, smoking status, systolic blood pressure, total and high-density lipoprotein cholesterol, age at diabetes diagnosis, HbA1c, and eGFR. **Conclusions**: If effective, DICTA could offer a scalable, digital-first approach for improving T2D management in primary care by combining patient-centered lifestyle coaching with real-time pharmacological clinical decision support.

## 1. Introduction

Type 2 diabetes (T2D), a major contributor to the global burden of non-communicable diseases, is responsible for nearly one million deaths annually [1,2,3,4]. It significantly increases the risk of cardiovascular disease and other serious complications [5,6,7]. Approximately 300,000 adults live with diabetes in Denmark, of whom 90% are diagnosed with T2D [8]. The majority of these individuals also experience comorbidities such as hypertension, dyslipidemia, and obesity, which affect over 80% of patients [9]. Despite significant advances in clinical care, many individuals struggle to achieve recommended treatment targets, adhere to lifestyle changes, or receive optimal pharmacological treatment [10,11]. These persistent challenges underscore the need for strategies to support patient engagement and self-management.

Lifestyle interventions, particularly those focused on weight reduction, have consistently shown the potential to improve glycemic control and even achieve remission in a significant portion of patients with T2D [1,2,3]. Although traditional face-to-face interventions can be effective [12,13,14], barriers such as transportation, time constraints, and personal preferences often hinder participation [15]. In contrast, digital health solutions, including eHealth (web-based) and mHealth (mobile applications) technologies, offer scalable, private, and personalized support with real-time feedback [16,17,18,19]. Evidence suggests that these technologies can promote behavior change and improve health outcomes, particularly when integrated into routine care and supported by professional guidance [20,21,22,23]. Despite the evidence, digital solutions are not implemented in most healthcare offerings, which may be due to lack of support and collaboration [20].

The Digital, Individualized, and Collaborative Treatment of Type 2 Diabetes in General Practice Based on Decision Aid (DICTA) study is a randomized controlled trial designed to evaluate the clinical impact of a comprehensive, novel, fully integrated dual digital intervention for newly diagnosed T2D patients in Danish primary care. This approach combines two synergistic components: a personalized eHealth lifestyle coaching application that supports behavioral changes based on patient-reported outcomes (PROs) [17,20,21,22,23], and a clinical decision support (CDS) system integrated within electronic health records to guide evidence-based pharmacological decisions. Drawing on prior research and stakeholder input, DICTA emphasizes patient involvement, real-time data sharing, and tailored care strategies for caregivers that, in combination, might drive the way to a long-term sustainable improvement not only in body weight but for cardiac risk factors as well [17,20,23,24]. To our knowledge, this has not been performed before. The study aims to determine whether this integrated digital approach can enhance patient engagement, support sustained behavior change, and improve health outcomes compared to usual care.

## 2. Materials and Methods

### 2.1. Study Design

**Figure 1:**
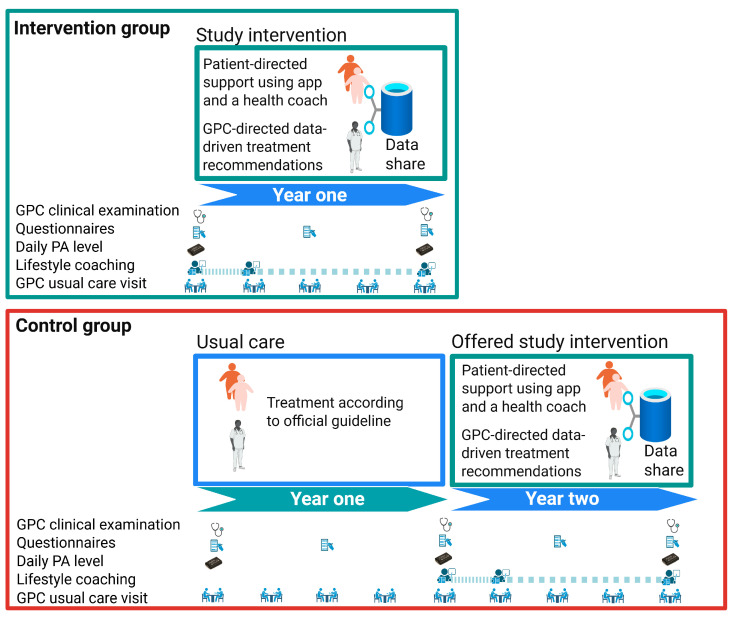
Overview of the DICTA RCT study, showing the different elements of the study. Created in BioRender. Nielsen, A. (2025) https://BioRender.com/1z45qf4 (accessed on 25 June 2025).

The DICTA study is a two-arm, cluster-randomized controlled trial (RCT) and will be conducted across multiple general practitioner clinics (GPCs) in Denmark. A randomization list will be generated using the program “sealedenvelope”, and cluster-randomization will occur at the GPC level, with an allocation ratio of 2:3 (intervention: control) and variable block sizes of 5 and 10 to ensure the balance between the two groups. Our allocation ratio of 2:3 is chosen based on an expectation that recruiting patients into the control group will be more challenging due to lower engagement incentives. Stratification will be based on two factors to ensure equal quality of care and no change in information sharing between study groups. Firstly, the GPC’s participation in the “Specialist supervised individualised multifactorial treatment of new clinically diagnosed type 2 diabetes in general practice (IDA): a prospective controlled multicentre open-label intervention study” [25] will be taken into consideration. Secondly, the physical address of the GPC will be considered. This is because some GPCs share address and health staff, and other GPCs operate as solo practices and thereby reduce the risk of intervention-based guidance of the control group patients. Other aspects such as age and gender of GPs, have not been taken into account as differences in quality of care is small and it is not possible to stratify on this. In Figure 1, the overview of the study’s elements is illustrated for both the intervention and control groups.

### 2.2. Population and Recruitment

A total of 400 individuals will be recruited through approximately 70 GPCs. Only clinics using the XMO electronic patient journal (EPJ) from CompuGroup Medical Denmark A/S will be eligible. To participate, GPCs must agree to integrate both the CDS tool and the LIVA e-health lifestyle coaching application into their EPJ system for management of T2D.

Eligible patients will be identified through the Danish Centre for Strategic Research in Type 2 Diabetes (DD2). The DD2 and GPCs will jointly facilitate the distribution of written and oral information about the DD2 and the DICTA study. Patients expressing interest will be contacted via email and telephone by a study team member within one to two weeks to confirm eligibility.

The inclusion criteria of the DICTA study are as follows: age between 18 and 80 years, T2D for less than five years (since diagnosis) or no other glucose-lowering medication than Metformin, HbA1c ≥ 53 mmol/mole (changed to 48 mmol/mole and approved by Ethics Committee at 13 July 2023), participation in DD2 and DICTA by the patient’s GPCs, possession of a smartphone, and no previous use of digital coaching via LIVA. The following criteria will be used to exclude patients from the study: participation in other clinical health-related trials, presence of serious or life-threatening disease, cognitive impairment, diseases such as dementia or diseases inhibiting communication, pregnancy, or active pursuance of the same, suspicion of eating disorder, or insufficient Danish language proficiency.

**Figure 2:**
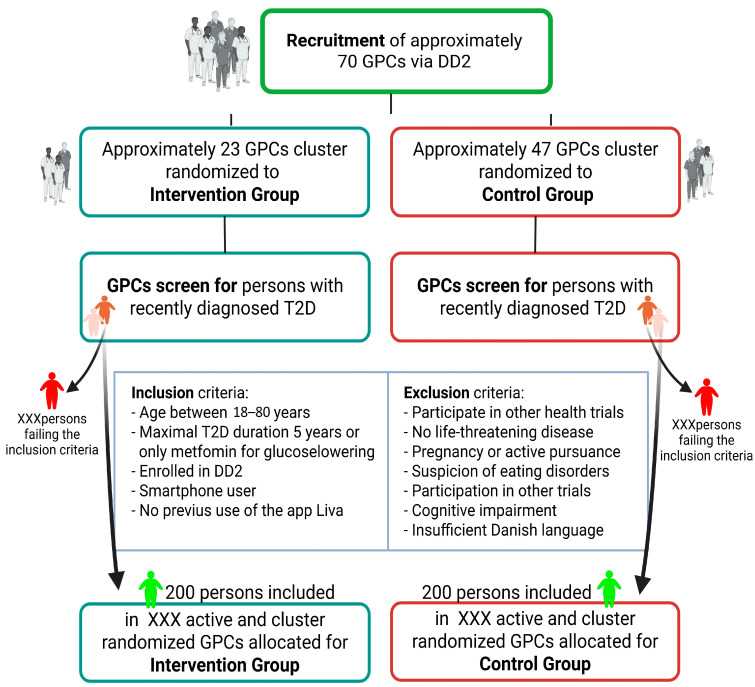
Flowchart of the expected patient flow of the randomized controlled trial. Created in BioRender. Nielsen, A. (2025) https://BioRender.com/15bsp7f (accessed on 25 June 2025).

Recruitment commenced in May 2021 and is expected to continue until 400 patients have consented to participate and initiated their involvement in the study. Figure 2 illustrates the expected patient flow in the DICTA RCT study. To keep up the inclusion rate, we planned from the start to continuously encourage GPCs from DD2 to join the study and to encourage participating GPCs to include more patients. Despite that, the inclusion rate was not as high as expected partly because of difficulties in meeting the initial inclusion criteria of HbA1c ≥ 53 mmol/mole because many of the patients were started early on Metformin, reducing HbA1c below 53 mmol/mole before inclusion in the study. Therefore, we reduced the inclusion criteria from HbA1c ≥ 53 mmol/mole to ≥48 mmol/mole in July 2023, which increased the inclusion rate.

### 2.3. Study Intervention

The DICTA intervention consists of two integrated components: a patient-directed eHealth lifestyle coaching program delivered via the LIVA application, and a clinician-directed CDS tool embedded into the EPJ system.

1. eHealth lifestyle coaching: Individuals with T2D in the intervention group will receive individualized digital coaching from a health coach through the LIVA application. PROs are shared with GPs and health staff via the EPJ, enabling tailored, data-driven lifestyle support.
2. CDS: GPs receive real-time, individualized, algorithm-based pharmacological treatment recommendations for managing T2D, hypertension, and hypercholesterolemia, as appropriate.

**Figure 3:**
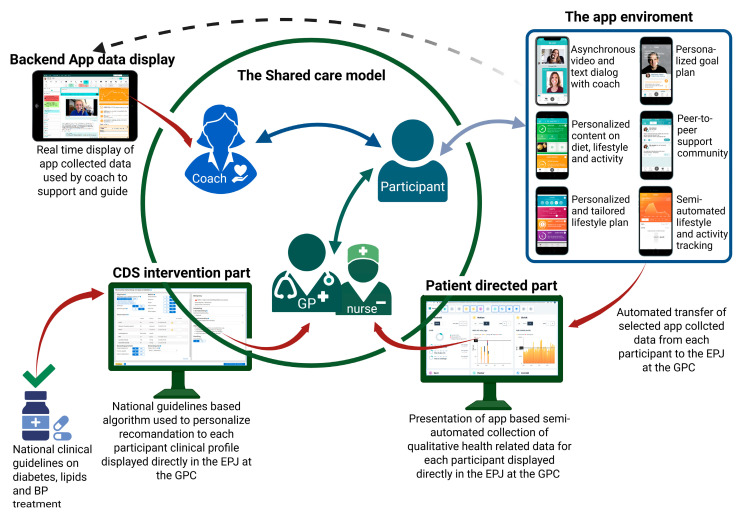
Visualizes how the intervention elements are interconnected and how the communication flows. Created in BioRender. Nielsen, A. (2025) https://BioRender.com/va1eruh (accessed 25 June 2025).

The intervention is customized to each participant’s personal health goals, which will form the foundation for digital health coaching. GPCs will be supported by the CDS system in making evidence-based pharmacological decisions. Health professionals access real-time patient data on lifestyle and goals, facilitating individualized care. Figure 3 shows how all of the elements in this study are interconnected.

### 2.4. eHealth Lifestyle Coaching

During the intervention, participants receive personalized eHealth lifestyle coaching from a personal health coach via the LIVA app, developed by Liva Healthcare A/S. The health coach will be educated as a dietician and in addition, have completed a 20-h course in digital coaching. At baseline, participants in the intervention group will be provided with instructions for installing the application on their smartphone or tablet, along with a link to schedule their initial virtual meeting with their health coach. If the app is not installed and/or a meeting is not scheduled, Liva Healthcare will proactively contact the participant via email and/or telephone to offer assistance.

Within two to four weeks after the baseline examination, participants will be scheduled for a digital motivational interview with their assigned health coach. Throughout the intervention period, participants receive ongoing, personalized, relationship-driven, asynchronous coaching sessions from the same health coach, fostering a continuous and supportive relationship. These coaching sessions occur on a weekly basis for the first three months of the intervention period, subsequently transitioning to fortnightly intervals for the following nine months.

The content of these coaching sessions is multifaceted, delivered through text and video formats, and is tailored to the patients’ specific goals and preferences. They incorporate customized content and opportunities for peer-to-peer support [26]. The coaching sessions address participants’ manually and automatically recorded data from wearables, smartphones, and/or tablets. These include information on goal achievement, dietary habits, physical activity, and lifestyle plans. Additionally, the app delivers notifications and reminders to encourage regular data entry and sustained engagement. All patient interaction and guidance within the digital coaching platform are systematically logged and monitored throughout the study to assess adherence. This includes tracking session completions, frequency of patient engagement, and response to coaching prompts, ensuring adherence despite the asynchronous delivery format. Health coaches have access to PROs, including step counts, interval walking data [27], and other personalized goals, which are displayed on the health coach’s computer or tablet. Algorithms within the app monitor behavioral trends and alert the health coach to significant changes, such as decreased physical activity, prompting timely intervention.

The health coaches are trained to support participants in formulating SMART (specific, measurable, agreed upon, realistic, and time-based) goals and developing action and coping plans, including the identification of barriers and problem-solving strategies. For example, a SMART goal might involve reaching 6000 steps daily, tracked via the participant’s smartphone. A comprehensive description of the LIVA application is provided in Supplementary Material, Table S1.

### 2.5. Clinical Decision Support for GPCs

GPs and health staff at participating GPCs will assess real-time PROs via the EPJ, enabling data-driven and individualized consultations. PROs, including physical activity, step count, dietary habits, goal attainment, and smoking status, are automatically transmitted from the patient’s smartphone via the LIVA application and displayed directly within the EPJ. This setup allows GPs and health staff to review lifestyle progress and discuss its implications for T2D management during routine visits.

The CDS tool, developed by Cambio Healthcare Systems A/S, Denmark, provides personalized pharmacological treatment recommendations within the EPJ. It is based on phenotyping and clinical algorithms and aligns with the most recent Danish Society for General Practice guidelines for the treatment of T2D. The CDS tool was co-developed with hospital specialists from Odense University Hospital, Slagelse Hospital, and Holbæk Hospital, and in consultation with GPs.

The CDS system incorporates key clinical parameters, including comorbidities, symptoms, and current medications, all extracted from the EPJ. These variables are processed using validated algorithms to generate evidence-based treatment guidance for hyperglycemia, hypertension, and hypercholesterolemia.

Within three months of inclusion, participants will be scheduled for a GPC visit to evaluate and, if necessary, adjust pharmacological treatment. If treatment targets for glycemic control, blood pressure, or lipid levels are not met, the GP will consider intensifying pharmacological treatment, based on recommendations provided by the CDS. The CDS prioritizes individualized pharmacological treatment to enhance treatment efficacy and minimize adverse effects.

### 2.6. Control Group

Participants assigned to the control group will receive usual care during the first year of the study period, in accordance with current Danish guidelines for the management of T2D. This care may include routine consultations with the GPs or other health staff, and all pharmacological treatment decisions will be made independently of the CDS system during this period.

Although the control group participants will not have access to the LIVA lifestyle coaching application or the CDS tool in the first year, they will undergo the same clinical examinations and complete the same questionnaires as those in the intervention group.

After 12 months, participants in the control group will be offered full intervention, mirroring the services and support provided to the intervention group in the first year.

### 2.7. Patient Examination and Data Sampling

Prior to the initial examination, all participants must provide signed, written informed consent. In Table 1, an overview of the timeline of patient examinations at GPCs and study components during the DICTA trial is provided.

The following parameters will be measured at baseline and at the 12-month follow-up visit: weight, systolic and diastolic blood pressure, and hip and waist circumference. However, height will only be measured at baseline. Blood pressure measurement will follow Danish clinical guidelines, which recommend measurements taken twice daily (morning and evening) over three consecutive days, prior to medication intake. Each session should include three repeated measurements. Whenever possible, these measurements should be conducted in the participant’s home. If the participant does not own a blood pressure monitoring device, one will be provided by the GPC. In both scenarios, the GPC will test and calibrate the blood pressure device during the baseline visit and record all subsequent measurements.

At both baseline and the 12-month follow-up, the following laboratory assessments will be performed: hemoglobin A1c (HbA1c), low-density lipoprotein (LDL) cholesterol from blood samples, and urine albumin/creatinine ratio (UACR) from spot urine samples. Availability of these data is contingent upon the participant’s written consent, which authorizes manual extraction from the EPJ. In Table 2, a detailed description of all the study variables and data sources is provided. All measurements will be conducted by trained clinical staff with prior instruction in standardized assessment techniques to ensure reliability and consistency.

Information regarding prescribed medications will be obtained from the Danish National Prescription Registry. Consent also allows access to the Shared Medication Record, from which information concerning the intended treatment dosage will be manually extracted at both baseline and follow-up.

At baseline, 6 months, and 12 months, the participant will receive a letter via e-Boks (a trusted Nordic provider of secure platforms and digital post boxes) containing a link to a questionnaire. The complete questionnaire battery, consisting of 45 items, is primarily composed of various standardized and validated instruments. For constructs where no validated questions were available, the research team has developed original items. These have been pilot-tested and revised accordingly before being included in the final questionnaire battery. The questionnaire battery covers topics including education level, physical activity, smoking habits, alcohol use, dietary habits, work ability (inspired by WPAI (Work Productivity and Activity Impairment Questionnaire [28])), general health (EQ-5D-5L and EQ-VAS [29]), mental well-being (SWEMWBS (Short Warwick-Edinburgh Mental Well-being scale [30,31])), and e-health literacy (eHLQ [32]-License number: E2019IA). The English version of the complete questionnaire battery is available in Supplementary Material, Table S2.

Much of the data will either be captured electronically or collected during the routine clinical consultations at the GPCs planned as part of the structured management of patients with T2D, thus minimizing the risk of missing data. However, if a GPC forgets to report data, we will have a very close and continuous collaboration with all the participating GPCs giving us the opportunity to make them aware of any forgetfulness. Finally, in case of missing data that cannot be recovered, we will use the “last carrying forward” or “the next carrying backwards” and supplement with sensitivity analyses excluding patients missing the data in focus.

## 3. Outcomes

The primary outcome is the one-year change in estimated ten-year cardiovascular risk assessed by SCORE2-Diabetes calculated from age, smoking status, systolic blood pressure, total and high-density lipoprotein cholesterol, age at diabetes diagnosis, HbA1c, and eGFR [33]. As we will be focusing on one-year changes in SCORE2-Diabetes, we do not consider it a problem that SCORE2-Diabetes does not include prevalent cardiovascular disease in the risk calculation as this variable will be constant over the one-year period on almost all of the patients. A previous Swedish study supports that these risk factors capture most of the residual cardiovascular risk in patients with T2D [34]. The secondary outcomes are group differences in changes between baseline and 12 months of follow-up in the following:

- Quality of life, measured by EQ-5D-5L;
- HbA1c, blood pressure, LDL cholesterol, smoking, and UACR;
- Use of glucose-lowering, antihypertensive, and lipid-lowering medications;
- Weight and abdominal circumference;
- Daily physical activity level, measured with AX3 accelerometers.

To support the assessment of the primary and secondary outcomes, a wide range of clinical, biochemical, and behavioral data will be collected throughout the study. Table 2 provides detailed descriptions of all study variables, including their definitions, measurement methods, data types, and sources. Details of the different ICD-10 codes included in this study are provided in Supplementary Material, Table S3.

## 4. Statistics

### 4.1. Power Calculations

In a recent publication using Danish health registries [35], we have calculated that the actual, absolute ten-year risk of cardiovascular disease for the entire DD2 cohort is 12.3%. Generally, 15% relative risk reduction is considered clinically relevant in clinical randomized controlled trials. Therefore, we define the minimal clinically relevant difference as 15% of 12.3%, which is approximately 2%. With a standard deviation of 4%, statistical power set at 90%, and a significance level of 5%, the study will require a sample size of at least 130 patients in both the intervention and the control group. Based on DD2 data [35], we expect approximately 20% of the participants to have prevalent cardiovascular disease and we anticipate 10–15% dropouts, therefore we will include 400 patients in total.

### 4.2. Analysis

In general, categorical variables will be summarized as numbers (%), continuous variables as min–max, mean (standard deviation, SD), and median (interquartile range, IQR), by randomization group.

The primary analysis of the primary outcome will follow an intention-to-treat principle, but due to the definition of the primary outcome, only participants with relevant measurement available at both baseline and one year of follow-up. We will employ a logistic regression model with robust variance estimation, accounting for GP clustering. As a secondary analysis approach, we will model the count of risk factors at both baseline and at one year of follow-up in a population-averaged longitudinal regression model based on generalized estimation equations (GEE), where group differences are included as a time-by-group interaction term. This approach accounts for clustering and potential missing measurements at each time point and will not be restricted to complete observations.

The analysis of the secondary outcomes will follow these two approaches outlined above. To generate hypotheses, several exploratory post hoc analyses will be conducted. The level for statistical significance is set at 5%. We will use statistical software such as StataBE (version 18.5 or newer) or R (version 4.5.1 or newer) for analysis.

## 5. Prospects and Potential for Upscaling

It is anticipated that DICTA will enhance the quality of care for individuals with T2D, promote positive lifestyle modifications, and be economically viable for society while reducing disparities in healthcare by improving the care of the most vulnerable and frail among individuals with T2D. Improved lifestyle has been demonstrated to positively impact the symptoms and prognosis of numerous other chronic diseases, including COPD, osteoarthritis, and hypertension. Consequently, it is anticipated that this will have a favorable influence on multi-morbidity. If effective, the intervention could be scaled up nationally through a collaboration between multiple Steno Diabetes Centres, universities, and GPCs. Moreover, the intervention has the potential to serve as a model for the management of other major chronic diseases in Denmark, with only minor program modifications. In the long term, the potential exists for international expansion of the DICTA study together with Compugroup Medical GmBh, Cambio Systems A/S, and Liva Healthcare A/S that are dominant players for digital patient and GP decision support in Northern Europe. Given the strong integration of CDS tools in Danish general practice and the centralized digital health infrastructure, generalizability to countries with less standardized systems may be constrained. Wider implementation would require contextual adaptation to local electronic health record systems, clinical workflows, and policy environments. Nonetheless, the conceptual model underlying DICTA—risk-based CDS combined with patient-centered digital engagement—remains broadly applicable and could be transferred with appropriate co-design and implementation planning. However, several factors must be considered when planning for national or international upscaling. First, variability in technology literacy among patients may influence both engagement and outcomes, particularly among older adults, individuals with lower educational attainment, or those with cognitive impairments. Second, differential levels of digital engagement, ranging from high interaction to minimal usage, could affect the intervention’s consistency and effectiveness across patient groups. Third, adherence and dropout rates may vary significantly in real-world settings without the structure and support of a research context. These challenges highlight the need for adaptive strategies, such as enhanced onboarding, digital literacy support, and continuous user feedback, to optimize equitable access and sustained impact at scale.

## 6. Ethics

The Regional Ethical Committee has approved the DICTA-RCT study (S-20190121) according to Danish law. The management and storage of participant data are being carried out in accordance with guidelines established by the Danish Data Protection Agency. The study has been duly registered and approved by Clinicaltrials.gov: NCT04880005.

There are no inherent risks to health and well-being associated with the engagement in the project. However, the potential for harm exists in the form of the development of eating disorders. Therefore, the dietician is directing particular attention to this matter and will exclude any patient if an eating disorder is suspected. Prior to enrollment, participants will be furnished with a comprehensive explanation of the study in both written and oral formats. Participation in the study and the database is voluntary, and individuals may withdraw at any time. The project has obtained permission from the Danish Data Protection Agency to store the data, and the data will be handled in accordance with the General Data Protection Regulation (GDPR). The Data Protection Officer at Steno Diabetes Center Odense and at Liva Healthcare will ensure protection and monitor data management, ensuring that it is in line with the established rules and regulations. Most of the study data will be securely stored within the Open Patient Data Explorative Network (OPEN) (https://open.rsyd.dk (accessed 25 June 2025), a custom-designed research database provided by the University of Southern Denmark. OPEN adheres to stringent requirements for data logging, password protection, and regular data backups, ensuring compliance with national legislation and protection against unauthorized access. Handwritten data and hard-copy source documents will be securely stored, protected against unauthorized access, and kept under lock, with access limited to authorized personnel only. The REDCap data collection system (Vanderbilt University, Nashville, TN, USA) will be utilized to manage the electronic reporting of source data, in accordance with established good clinical practice standards. During the GP examination, measurements will be entered directly into REDCap, where GPC will be responsible for filling out a designated questionnaire. Access to REDCap will be granted to GPCs via a link to a secure login page. Liva Healthcare is in compliance with GDPR.

## Figures and Tables

**Table 1:** Timeline of patient examinations in general practice and study components during the DICTA trial.

Time Point	Study Component	Intervention Group	Control Group
Recruitment	Information and consent	Oral and written information provided by the GP	Oral and written information provided by the GP
Baseline visit	Written informed consent	Written informed consent signed by the participant	Written informed consent signed by the participant
	DICTA examination ^1^	Full clinical examination: blood/urine sampling, home BP, height (baseline only), weight, waist/hip circumference Accelerometer setup	Full clinical examination: blood/urine sampling, home BP, height (baseline only), weight, waist/hip circumference Accelerometer setup
	DICTA questionnaire ^2^	Questionnaire on lifestyle, quality of life, and eHealth literacy	Questionnaire on lifestyle, quality of life, and eHealth literacy
	DD2 enrollment (if not enrolled already)	DD2 Blood and urine samples + DD2 online questionnaire	DD2 Blood and urine samples + DD2 online questionnaire
Week 1–2 post baseline	eHealth lifestyle coaching onboarding	Digital motivational interview, app installation support, goal setting with health coach (via LIVA app version 4.19.0 or older for IOS and version 4.10.83 or older for Android)	NA
Every 1–3 months	Follow-up visits ^3^ (if risk factors unmet)	CDS-supported medical review (hyperglycemia, hypertension, and hypercholesterolemia) Lifestyle check-in via LIVA, review of PRO	NA
12-month visit	DICTA examination ^1^	Full clinical examination: blood/urine sampling, home BP, weight, waist/hip circumference Accelerometer setup	Full clinical examination: blood/urine sampling, home BP, weight, waist/hip circumference Accelerometer setup
	DICTA questionnaire ^2^	Questionnaire on lifestyle, quality of life (EQ-5D-5L), and eHealth literacy	Questionnaire on lifestyle, quality of life, and eHealth literacy
	CDS medication adjustment	Based on updated PRO and guideline-based CDS recommendations via EPJ	NA

^1^ DICTA examination: includes physical measures (weight, waist/hip circumference, and height [baseline only]), home BP monitoring, and accelerometer-based activity tracking. ^2^ DICTA Questionnaire: administered via secure digital post (e-Boks). ^3^ Follow-up visits are initiated only if treatment targets are unmet, as per Danish guidelines and CDS alerts. DD2: The Danish Centre for Strategic Research in Type 2 Diabetes, NA: not applicable, LIVA: Lifestyle InterVention App, CDS: Clinical Decision Support, and PRO: Patient-reported outcomes.

**Table 2:** Study variables and data sources.

Category	Variable	Definition/Measurement	Type	Source
Clinical Measurements	Height	Measured without shoes (cm)	Continuous	DICTA GP Questionnaire (REDCap)
	Weight	Measured with clothes but without shoes, (−1 kg adjustment) (kg)	Continuous	DICTA GP Questionnaire (REDCap)
	Waist Circumference	Horizontal circumference at midpoint between the lowest rib and upper point of iliac crest (cm) If the ribs and the iliac crest are not accessible, the horizontal circumference just above the navel	Continuous	DICTA GP Questionnaire (REDCap)
	Hip Circumference	Horizontal circumference at iliac crest or one handbreadth above inguen (cm) If the ribs and the iliac crest are not accessible: horizontal circumference at the level of a handbreadth above the inguen	Continuous	DICTA GP Questionnaire (REDCap)
	Blood Pressure	Systolic and diastolic home BP using calibrated monitor (mmHg)	Continuous	DICTA GP Questionnaire (REDCap)
Biochemical Measurements	HbA1c	Fasting venous blood sample from the arm. Analyzed by the clinical biochemistry department associated with the GP (mmol/mole)	Continuous	EPJ
	LDL Cholesterol	Fasting venous blood sample from the arm. Analyzed by the clinical biochemistry department associated with the GP (mmol/L)	Continuous	EPJ
	Urine Albumin/Creatinine Ratio (UACR)	Fasting urine sample (mg/g)	Continuous	EPJ
Lifestyle Indicators	Smoking, Physical Activity, Alcohol, Diet	Self-reported via questionnaire	Categorical	DICTA Questionnaire (Table S2)
Quality of Life	EQ-5D-5L	Standardized measure of health-related quality of life	Categorical	DICTA Patient Questionnaire (Table S2)
Mental health	SWEMWBS	Standardized measure of mental well-being	Categorical	DICTA Patient Questionnaire (Table S2)
Work ability	WPAI	Standardized measure of work ability	Categorical	DICTA Patient Questionnaire (Table S2)
Technology	eHLQ	Standardized measure of e-health literacy	Categorical	DICTA Patient Questionnaire (Table S2)
Physical Activity Patterns	Daily Physical Activity - Steps - Sleep - Intensities - Walking - Running - Standing - Sitting - Moving	Measured by accelerometers (Axivity AX3, NewCastle)	Continuous	Accelerometers AX3 and data downloaded by a software program called OmGui (version 1.0.0.45)
	Daily Step Count	Number of steps via smartphone integration	Continuous	LIVA App
Health Indicators	Medications	Prescribed pharmaceuticals dispensed for each patient in relation to the use of hypertension, hypercholesterolemia, and glucose-lowering drugs, T2D, and hypertension, cholesterol	Continuous	Danish National Prescription Registry
	Healthcare Utilization	Frequency of medical visits, hospitalizations	Categorical/Count	Danish National Patient Register

DICTA questionnaire for GPs; GP, General practice; REDCap, Research Electronic Data Capture (Vanderbilt University, Nashville, TX, USA); HbA1c, Glycated hemoglobin; LDL, low-density lipoprotein; LIVA, Lifestyle InterVention App; DICTA questionnaire on quality of life, lifestyle, and eHealth literacy as described in Table S2.

## Data Availability

Data are not available because this is a protocol for a RCT study. Request to access the data that this study will generate can be made to Jens Søndergaard.

## Supplementary Materials

The following supporting information can be downloaded at: https://www.mdpi.com/article/10.3390/nu17152494/s1, Table S1: Template of the intervention description and replication checklist for the eHealth lifestyle coaching tool (LIVA) [26]. Table S2: Questionnaire. Table S3: Overview of ICD-10 disease classifications used in the present study.
