# Supplementary material for: Protocol for the Digital, Individualized, and Collaborative Treatment of Type 2 Diabetes in General Practice Based on Decision Aid (DICTA)—A Randomized Controlled Trial
Source: Nutrients. 2025 Jul 30;17(15):2494. doi: 10.3390/nu17152494 (PMC12348545; doi:10.3390/nu17152494)
Supplement: Supplementary file 1 [file nutrients-17-02494-s001.zip › nutrients-3749574-supplementary.pdf]

Supplementary Materials

Table S1. Template of the intervention description and replication checklist for the eHealth lifestyle coaching tool (LIVA) [1].

Item	*Description*
eHealth coaching sessions	Prior to the intervention, the health coaches receive training in setting SMART (specific, measurable, agreed upon, realistic, and time-based) goals with the participants using the eHealth solution LIVA, and in setting up action and coping plans that address barrier identification and problem solving. Participants in the intervention group have one digital meeting with their health coach, followed by 25 asynchronous web-based consultations, based on dialog, by means of text or video for the first 12 months. The consultations address the participant's registrations, goal setting, and questions regarding diet, exercise, and lifestyle plans, taking into consideration chronic diseases. The LIVA app is set up with short explanations about different functions and notifications, and reminders for the participants to register and give feedback about the health coaching. The sessions provide participants with information about their status, a specific focus on goals, and recommendations on how to improve their behaviors.<br>Include behavior change technique from Coventry, Aberdeen, and London-Refined taxonomy (hereafter referred to as BCT) as follows: provide information on the consequences of the behavior, in general, and to the individual goal setting, behavior and outcome, action planning, and barrier identification/problem solving; set graded tasks; prompt review of behavioral goals; prompt review of outcome goals; prompt rewards contingent on effort or progress toward behavior; prompt generalization of a target behavior; and provide feedback on performance (supplementary material).
Goals and inputs	Goals and inputs are always driven by the participant and are available to the participant, who can choose the focus area, set specific goals, and keep a record of specified behaviors by reporting them on a daily, weekly, or monthly basis. This allows the user and the health coach to follow progress or setbacks as the numbers and registrations are visualized using graphs and curves. All coaching by the health coach follows national guidelines from the Danish National Board of Health.
Dietary goals and plans	Dietary goals and plans can be set at many different levels (e.g., from simple changes aiming at changing one meal a day to more complex changes aiming at a completely new diet for the remedying of digestion problems).
Physical activity goals and plans	Physical activity goals and plans involve goal setting and recording of the type of physical activity and time for executing the given physical activity. The participant receives advice and/or a video on activities in a variety of contexts to foster physical activity as a more integrated part of life (BCT: provide instruction on how to perform the behavior, prompt generalization of a target behavior, and provide relapse prevention/coping planning).
Life goals	Goals on a healthy, joyful life as the participant sees it (e.g., daily life with less stress, stronger social bonds with friends and family, coping skills for diseases, etc.).
Weight	Set the current weight and goal for a lower or higher weight and register new measurements on a daily, weekly, or monthly basis.
Steps	When downloading the LIVA app, the participant can accept direct import of their information on steps recorded on a smartphone, and tailored messages on progress toward a set goal

	appear simultaneously (BCT: teach-to-use prompts/cues). Step count monitoring is encouraged but not required to enter the LIVA study. Some participants will have other ways of registering their physical activity level.
Pain, sleep, and mood	Give daily feedback on pain, sleep, and mood, which can affect the ability to perform a given behavior (BCT: relapse prevention/coping planning).
Smoking	Set goals to bring down the number of cigarettes smoked on a daily basis, leading to cessation.
Blood glucose and blood pressure	Keep a record of specified measures expected to be influenced by the different behavior changes addressed. In LIVA, this includes blood glucose and blood pressure measurements (BCT: prompt self-monitoring of behavioral outcomes and provide information on consequences of the behavior, in general, and for the individual).
Forum	An online forum where the participant can exchange knowledge, gain social support, and build new relationships, and the health coach can provide advice to the participant (BCT: plan social support/change).
Coaching providers	Health coaches with basic training as nurses, physiotherapists, dieticians, or occupational therapists. In Denmark, all four education types consist of 420 European Credit Transfer System (ECTS) Points (3.5 years of full-time education). In addition to their education as health care professionals, they all undergo special training in using digital health coaching and practice digital health coaching for at least 2 years.
Coaching approach	Individually delivered via the app or web-based delivery.
Coaching location	Initial personal meetings in municipality health centers, general practice medical centers, or the research unit for general practice at the University of Southern Denmark, or over the internet, and then, solely web-based delivery.
Coaching time and quantity	The initial consultation with a health coach is estimated to last for approximately 45-60 minutes. The subsequent asynchronous eHealth coaching sessions are carried out once a week in the first 3 months and then every other week for the last 9 months. Thereafter, the participant could receive monthly eHealth coaching sessions and use LIVA as a personal BCT tool (BCT: use of follow-up prompts) for 12 months.
Tailoring	Every participant receives personalized eHealth coaching sessions from their designated health coach. The feedback provided is based on the participant's inputs on LIVA.

Table S2. Questionnaire

The questionnaire was sent out to the participants every six months and contained the following aspects.

Background section – self-developed / standard demographic questions	
**The Question asked in Danish**	**The English version**
Hvad er dit højest gennemførte uddannelsesniveau?<br>- Ingen videregående uddannelse<br>- Kort videregående uddannelse på 2 år (eks. Erhvervsakademi)<br>- Mellemlang uddannelse på 3-4,5 år (eks. Professionsbachelor)<br>- Lang videregående uddannelse på minimum 5 år (eks. Kandidat og PhD på universitetet)<br>- Ved ikke	What is your highest completed level of education?<br>- No higher education<br>- Short higher education of 2 years (e.g., Academy Profession)<br>- Medium-length education of 3–4.5 years (e.g., Professional Bachelor)<br>- Long higher education of at least 5 years (e.g., Master's degree and PhD at a university)<br>- Do not know
Hvad er din civilstatus?<br>- Gift eller registreret partnerskab<br>- Ugift<br>- Enke eller længst levende partner<br>- Skilt eller opbrud registreret partnerskab	What is your marital status?<br>- Married or in a registered partnership<br>- Single<br>- Widow or surviving partner<br>- Divorced or dissolved registered partnership
Hvad er din beskæftigelsesstatus?<br>- Tilknyttet arbejdsmarkedet (ansat eller selvstændig)<br>- Arbejdsløs (barselsorlov eller dagpenge)<br>- Arbejdsløs (kontanthjælp)<br>- Studerende<br>- Førtidspensioneret<br>- Pensioneret	What is your employment status?<br>- Attached to the labor market (employed or self-employed)<br>- Unemployed (maternity leave or unemployment benefits)<br>- Unemployed (social assistance)<br>- Student<br>- Early retirement pensioner<br>- Retired

Mental well-being – SWEMWBS		
**The Question asked in Danish**	**The original English version**	**SWEMWBS Item**
Jeg har følt mig optimistisk i forhold til fremtiden<br>- På intet tidspunkt<br>- Sjældent<br>- Noget af tiden<br>- Ofte<br>- Hele tiden	I've been feeling optimistic about the future<br>- None of the time<br>- Rarely<br>- Some of the time<br>- Often<br>- All of the time	I've been feeling optimistic about the future
Jeg har følt mig nyttig<br>- På intet tidspunkt<br>- Sjældent<br>- Noget af tiden<br>- Ofte<br>- Hele tiden	I've been feeling useful<br>- None of the time<br>- Rarely<br>- Some of the time<br>- Often<br>- All of the time	I've been feeling useful
Jeg har følt mig afslappet<br>- På intet tidspunkt	I've been feeling relaxed<br>- None of the time	I've been feeling relaxed

- Sjældent<br>- Noget af tiden<br>- Ofte<br>- Hele tiden	- Rarely<br>- Some of the time<br>- Often<br>- All of the time	
Jeg har klaret problemer godt<br>- På intet tidspunkt<br>- Sjældent<br>- Noget af tiden<br>- Ofte<br>- Hele tiden	I’ve been dealing with problems well<br>- None of the time<br>- Rarely<br>- Some of the time<br>- Often<br>- All of the time	I’ve been dealing with problems well
Jeg har tænkt klart<br>- På intet tidspunkt<br>- Sjældent<br>- Noget af tiden<br>- Ofte<br>- Hele tiden	I’ve been thinking clearly<br>- None of the time<br>- Rarely<br>- Some of the time<br>- Often<br>- All of the time	I’ve been thinking clearly
Jeg har følt mig tæt på andre mennesker<br>- På intet tidspunkt<br>- Sjældent<br>- Noget af tiden<br>- Ofte<br>- Hele tiden	I’ve been feeling close to other people<br>- None of the time<br>- Rarely<br>- Some of the time<br>- Often<br>- All of the time	I’ve been feeling close to other people
Jeg har været i stand til at danne min egen mening om ting<br>- På intet tidspunkt<br>- Sjældent<br>- Noget af tiden<br>- Ofte<br>- Hele tiden	I’ve been able to make up my own mind about things<br>- None of the time<br>- Rarely<br>- Some of the time<br>- Often<br>- All of the time	I’ve been able to make up my own mind about things

Health status – EQ-5D-5L + EQ-VAS (EuroQoL Group)

**The Question asked in Danish**	**The original English version**	**EQ-5D-5L Item**
Bevægelighed<br>- Jeg har ingen problemer med at gå omkring<br>- Jeg har få problemer med at bevæge mig omkring<br>- Jeg har moderate problemer med at bevæge mig omkring<br>- Jeg har alvorlige problemer med at bevæge mig omkring<br>- Jeg er ikke i stand til at bevæge mig omkring	Mobility<br>- I have no problems in walking about<br>- I have slight problems in walking about<br>- I have moderate problems in walking about<br>- I have severe problems in walking about<br>- I am unable to walk about	Mobility
Selvpleje/egenomsorg<br>- Jeg har ingen problemer med at vaske og klæde mig selv på<br>- Jeg har få problemer med at vaske og klæde mig selv på	Self-care<br>- I have no problems washing or dressing myself<br>- I have slight problems washing or dressing myself	Self-care

- Jeg har moderate problemer med at vaske og klæde mig selv på<br>- Jeg har alvorlige problemer med at vaske og klæde mig selv på<br>- Jeg er ikke i stand til at vaske og klæde mig selv på	- I have moderate problems washing or dressing myself<br>- I have severe problems washing or dressing myself<br>- I am unable to wash or dress myself	
Sædvanelige aktivitetsniveau (fx arbejde, studie, husholdning, familie og fritidsaktiviteter)<br>- Jeg har ingen problemer med at udføre min sædvanlige aktivitetsniveau sædvanlige aktivitet<br>- Jeg har få problemer med at udføre min sædvanlige aktivitet<br>- Jeg har moderate problemer med at udføre min sædvanlige aktivitet<br>- Jeg har alvorlige problemer med at udføre min sædvanlige aktivitet<br>- Jeg er ikke i stand til at udføre min sædvanlige aktivitet	Usual activities (e.g., work, study, housework, family, or leisure activities)<br>- I have no problems doing my usual activities<br>- I have slight problems doing my usual activities<br>- I have moderate problems doing my usual activities<br>- I have severe problems doing my usual activities<br>- I am unable to do my usual activities	Usual activities
Smerte / ubehag<br>- Jeg mærker ingen smerte/ubehag<br>- Jeg mærker lidt smerte/ubehag<br>- Jeg mærker moderat smerte/ubehag<br>- Jeg mærker alvorlig smerte/ubehag<br>- Jeg mærker ekstrem smerte/ubehag	Pain / discomfort<br>- I have no pain or discomfort<br>- I have slight pain or discomfort<br>- I have moderate pain or discomfort<br>- I have severe pain or discomfort<br>- I have extreme pain or discomfort	Pain/discomfort
Angst / depression<br>- Jeg føler mig ikke angst eller deprimeret<br>- Jeg føler mig lidt angst eller deprimeret<br>- Jeg føler mig moderat angst eller deprimeret<br>- Jeg føler mig alvorligt angst eller deprimeret<br>- Jeg føler mig ekstremt angst eller deprimeret	Anxiety / depression<br>- I am not anxious or depressed<br>- I am slightly anxious or depressed<br>- I am moderately anxious or depressed<br>- I am severely anxious or depressed<br>- I am extremely anxious or depressed	Anxiety/depression
Hvor godt føler du dit helbred er i dag (0–100 skala)	How good is your health today? (0–100 scale)	EQ-VAS

Smoking habits – self-developed

The Question asked in Danish	The English translation
Er du ryger?<br>- Ja	Do you smoke?<br>- Yes

- Nej	- No
Hvor mange cigaretter ryger du i gennemsnit om ugen?	How many cigarettes do you smoke per week on average?
I hvor høj grad er du motiveret for at ændre dine rygevaner?<br>- Slet ikke<br>- Lidt<br>- I nogen grad<br>- En del<br>- Meget	How motivated are you to change your smoking habits?<br>- Not at all<br>- A little<br>- To some extent<br>- Quite a bit<br>- Very much

Alcohol consumption – self-developed

**The Question asked in Danish**	**The English translation**
Hvor mange genstande drikker du på ugentlig basis?	How many units of alcohol do you drink per week?
I hvor høj grad er du motiveret for at ændre dine alkoholvaner?<br>- Slet ikke<br>- Lidt<br>- I nogen grad<br>- En del<br>- Meget	How motivated are you to change your alcohol habits?<br>- Not at all<br>- A little<br>- To some extent<br>- Quite a bit<br>- Very much

Diet habits – self-developed

**The Question asked in Danish**	**The English translation**
Hvor hyppigt spiser du grøntsager og/eller rodfrugter?<br>- To gange dagligt eller oftere<br>- En gang dagligt<br>- Nogle gange i løbet af ugen<br>- En gang i ugen eller sjældnere	How often do you eat vegetables and/or root vegetables?<br>- Twice daily or more often<br>- Once daily<br>- Several times during the week<br>- Once a week or less often
Hvor hyppigt spiser du frugt og/eller bær?<br>- To gange dagligt eller oftere<br>- En gang dagligt<br>- Nogle gange i løbet af ugen<br>- En gang i ugen eller sjældnere	How often do you eat fruit and/or berries?<br>- Twice daily or more often<br>- Once daily<br>- Several times during the week<br>- Once a week or less often
Hvor hyppigt spiser du fisk eller skaldyr som hovedret?<br>- Tre gange om ugen eller oftere<br>- To gange om ugen<br>- En gang om ugen<br>- Et par gange om måneden eller sjældnere	How often do you eat fish or shellfish as a main course?<br>- Three times a week or more often<br>- Twice a week<br>- Once a week<br>- A couple of times a month or less often
Hvor hyppigt spiser du wienerbrød, chokolade, slik og/eller drikker sodavand?<br>- Dagligt<br>- Næsten hver dag	How often do you eat pastries, chocolate, candy, and/or drink soft drinks?<br>- Daily<br>- Almost every day

- Et par gange om ugen<br>- En gang om ugen eller sjældnere	- A couple of times a week<br>- Once a week or less often
I hvor høj grad er du motiveret for at ændre dine kostvaner?<br>- Slet ikke<br>- Lidt<br>- I nogen grad<br>- En del<br>- Meget	How motivated are you to change your dietary habits?<br>- Not at all<br>- A little<br>- To some extent<br>- Quite a bit<br>- Very much

Physical activity – self-developed

**The Question asked in Danish**	**The English translation**
Hvor meget tid bruger du om ugen på fysisk træning, der gør dig forpustet?<br>- Ingen tid<br>- Mindre en 0.5 time<br>- 0-5-1 time<br>- 1-2 timer<br>- 2 timer eller mere	How much time per week do you spend on physical training that makes you short of breath?<br>- No time<br>- Less than 0.5 hour<br>- 0-5-1 hour<br>- 1-2 hours<br>- 2 hours or more
Hvor meget tid bruger du om ugen på hverdagsmotion?<br>- Ingen tid<br>- Mindre en 0.5 time<br>- 0-5-1 time<br>- 1-1.5 time<br>- 1.5-2.5 time<br>- 2.5-5 timer<br>- 5 timer eller mere	How much time per week do you spend on everyday physical activity?<br>- No time<br>- Less than 0.5 hour<br>- 0-5-1 hour<br>- 1-1.5 hours<br>- 1.5-2.5 hours<br>- 2.5-5 hours<br>- 5 hours or more
I hvor høj grad er du motiveret for at ændre på dine motionsvaner?<br>- Slet ikke<br>- Lidt<br>- I nogen grad<br>- En del<br>- Meget	How motivated are you to change your exercise habits?<br>- Not at all<br>- A little<br>- To some extent<br>- Quite a bit<br>- Very much

Work ability and absenteeism – inspired by WPAI

**The Question asked in Danish**	**The original English version**
Er du i øjeblikket lønmodtager?<br>- Ja<br>- Nej	Are you currently employed (as a wage earner)?<br>- Yes<br>- No
I løbet af de sidste 7 dage, hvor mange dage udeblev du fra arbejde grundet andre årsager (ferie/studie)?<br>- 0<br>- 1<br>- 2<br>- 3<br>- 4	How many days in the past 7 did you miss work for other reasons (e.g., vacation, study participation)?<br>- 0<br>- 1<br>- 2<br>- 3<br>- 4<br>- 5

- 5<br>- 6<br>- 7	- 6<br>- 7
I løbet af de sidste 7 dage, hvor mange timer har du udeblevet fra arbejde grundet helbredsproblemer?	How many hours in the past 7 days did you miss work due to health problems?
I løbet af de sidste 7 dage, hvor mange timer har du været på arbejde?	How many hours have you worked in the past 7 days?
I løbet af de sidste 7 dage, hvor meget påvirkede dit helbred din produktivitet på arbejdet?<br>- 1<br>- 2<br>- 3<br>- 4<br>- 5<br>- 6<br>- 7<br>- 8<br>- 9<br>- 10	How much did your health affect your productivity at work in the past 7 days?<br>- 1<br>- 2<br>- 3<br>- 4<br>- 5<br>- 6<br>- 7<br>- 8<br>- 9<br>- 10
I løbet af de sidste 7 dage, hvor meget påvirkede dit helbred din evne til daglige aktiviteter?<br>- 1<br>- 2<br>- 3<br>- 4<br>- 5<br>- 6<br>- 7<br>- 8<br>- 9<br>- 10	How much did your health affect your ability to perform daily activities in the past 7 days?<br>- 1<br>- 2<br>- 3<br>- 4<br>- 5<br>- 6<br>- 7<br>- 8<br>- 9<br>- 10

Digital Health Literacy – eHLQ

**The Question asked in Danish**	**The original English version**	**eHLQ Domain**
Teknologi gør, at jeg føler mig engageret i min egen sundhed<br>- Meget uenig<br>- Uenig<br>- Enig<br>- Meget enig	Technology makes me feel engaged in my own health<br>- Strongly disagree<br>- Disagree<br>- Agree<br>- Strongly agree	eHLQ 1: Using technology to process health information
Jeg bruger teknologi til at finde information om sundhed<br>- Meget uenig<br>- Uenig<br>- Enig<br>- Meget enig	I use technology to find health information<br>- Strongly disagree<br>- Disagree<br>- Agree<br>- Strongly agree	eHLQ 1

Jeg bruger ofte teknologi til at forstå problemstillinger om mit helbred<br>- Meget uenig<br>- Uenig<br>- Enig<br>- Meget enig	I often use technology to understand issues related to my health<br>- Strongly disagree<br>- Disagree<br>- Agree<br>- Strongly agree	eHLQ 1
Teknologi hjælper mig med at vælge sundhedstilbud, der er bedst for mig<br>- Meget uenig<br>- Uenig<br>- Enig<br>- Meget enig	Technology helps me choose the best health services for me<br>- Strongly disagree<br>- Disagree<br>- Agree<br>- Strongly agree	eHLQ 6: Access to digital health services that work
Jeg synes, teknologi hjælper mig til at tage mig af mit helbred<br>- Meget uenig<br>- Uenig<br>- Enig<br>- Meget enig	I believe technology helps me manage my health<br>- Strongly disagree<br>- Disagree<br>- Agree<br>- Strongly agree	eHLQ 1
Jeg bruger teknologi til at dele informationer om mit helbred<br>- Meget uenig<br>- Uenig<br>- Enig<br>- Meget enig	I use technology to share information about my health<br>- Strongly disagree<br>- Disagree<br>- Agree<br>- Strongly agree	eHLQ 3: Ability to actively engage with digital services
Jeg får bedre hjælp fra sundhedsprofessionelle, når jeg bruger teknologi<br>- Meget uenig<br>- Uenig<br>- Enig<br>- Meget enig	I receive better support from health professionals when using technology<br>- Strongly disagree<br>- Disagree<br>- Agree<br>Strongly agree	eHLQ 7: Digital services that suit individual needs
Jeg bruger teknologi til at holde styr på mine sundhedsoplysninger<br>- Meget uenig<br>- Uenig<br>- Enig<br>- Meget enig	I use technology to track my health data<br>- Strongly disagree<br>- Disagree<br>- Agree<br>- Strongly agree	eHLQ 2: Understanding of health concepts and language
Teknologi forbedrer min kommunikation med sundhedsprofessionelle	Technology improves my communication with health professionals	eHLQ 4: Feel safe and in control

- Meget uenig<br>- Uenig<br>- Enig<br>- Meget enig	- Strongly disagree<br>- Disagree<br>- Agree<br>- Strongly agree	
Teknologi er nyttigt til at følge min sundhedstilstand<br>- Meget uenig<br>- Uenig<br>- Enig<br>- Meget enig	Technology is useful for monitoring my health status<br>- Strongly disagree<br>- Disagree<br>- Agree<br>- Strongly agree	eHLQ 5: Motivated to engage with digital services

Table S3. Overview of ICD-10 disease classifications used in the present study

<table>
<tr><td colspan="2">Fatal cardiovascular disease–cause-specific mortality due to any of the following:
Hypertensive disease: ICD-10 codes I10-16, ICD-9 codes 401-405
Ischemic heart disease: ICD-10 codes I20-25, ICD-9 codes 410-414
Arrhythmias, heart failure: ICD-10 codes I46-52, ICD-9 codes 426-429
Cerebrovascular disease: ICD-10 codes I60-69, ICD-9 codes 430-438
Atherosclerosis/aortic aneurysm: ICD-10 codes I70-73, ICD-9 codes 440-443
Sudden death and death within 24 hours of symptom onset: ICD-10 codes R96.0-R96.1, ICD-9 codes 798.1, 798.2

Endpoints excluded from the above endpoint:
Myocarditis, unspecified: ICD-10 code I51.4, ICD-9 code 426.7
And endpoints excluded below</td></tr>
<tr><td colspan="2">Non-fatal cardiovascular disease:
Non-fatal myocardial infarction: ICD-10 codes I21-I23, ICD-9 code 410
Non-fatal stroke: ICD-10 codes I60-69, ICD-9 codes 430-438

Excluded from the non-fatal stroke endpoint:
Subarachnoid hemorrhage: ICD-10 code I60, ICD-9 code 429
Subdural hemorrhage: ICD-10 code I62, ICD-9 code 430
Cerebral aneurysm: ICD-10 code I67.1, ICD-9 code 432.1
Cerebral arteritis: ICD-10 code I68.2, ICD-9 code 437.3
Moyamoya: ICD-10 code I67.5, ICD-9 code 437.4</td></tr>
<tr><td>Variables</td><td>Definition</td></tr>
<tr><td>All-cause mortality</td><td>Death from any cause</td></tr>
<tr><td>5-point Major Adverse Cardiovascular Event (MACE)</td><td>First occurrence of non-fatal myocardial infarction, non-fatal stroke, coronary revascularization, heart failure hospitalization, and cardiovascular death. (See definitions below).</td></tr>
<tr><td>Myocardial infarction</td><td>DI21</td></tr>
<tr><td>Stroke</td><td>DI61, DI63, DI64</td></tr>
<tr><td>Coronary revascularization</td><td>KFNA, KFNB, KFNC, KFND, KFNE, KFNF, KFNG, KFNH20</td></tr>
<tr><td>Heart failure (hospitalization)</td><td>DI50, DI110, DI130, DI132</td></tr>
<tr><td>Cardiovascular death</td><td>DI00-DI99 from Danish Registry of Causes of Death OR death from The Civil Registration System AND within 30 days a diagnosis of the following:
Myocardial infarction: DI21,
Unstable Angina: DI200,
Stroke: DI61, DI63, DI64,
Heart failure hospitalization: DI50, DI110, DI130, DI132,
Coronary revascularization: KFNA, KFNB, KFNC, KFND, KFNE, KFNF, KFNG, KFNH20,
Peripheral revascularization: Thrombectomy or embolectomy: KPDE, KPEE, KPFE
Thromboendarterectomy: KPDF, KPEF, KPFF, KPDU74, KPEU74, KPFU74
Bypass-operations: KPDH, KPEH, KPFH, KPGH20+21+22+23+30+31+40+99
Angioplasty: KPDN, KPEN, KPFN KPDU82, KPEU82, KPFU82
Percutaneous angioplasty: KPDP, KPEP, KPFP, KPDU83, KPEU83, KPFU83
Unspecified: KPDW, KPEW, KPFW,
Complications after myocardial infarction (DI24)</td></tr>
</table>

	Chronic ischemic heart disease (DI25)<br>Cardiac arrest/sudden cardiac death (DI46)<br>Ventricular tachycardia (DI472)<br>Ventricular fibrillation/flutter (DI490)

Reference

1. Brandt, C.J., et al., *Evaluation of the Clinical and Economic Effects of a Primary Care Anchored, Collaborative, Electronic Health Lifestyle Coaching Program in Denmark: Protocol for a Two-Year Randomized Controlled Trial.* JMIR Res Protoc, 2020. **9**(6): p. e19172.
